# Toward a push–pull strategy against invasive snails using chemical and visual stimuli

**DOI:** 10.1038/s41598-024-62225-6

**Published:** 2024-05-20

**Authors:** Cédric Kosciolek, Gaylord A. Desurmont, Thierry Thomann, Alberto Zamprogna, Valérie Caron

**Affiliations:** 1 CSIRO European Laboratory, Campus International de Baillarguet, 34980 Montferrier-sur-Lez, France; 2 European Biological Control Laboratory, USDA-ARS, 810 Avenue de Baillarguet, 34980 Montferrier-sur-Lez, France; 3 CSIRO Health and Biosecurity, Clunies Ross Street, Acton, ACT 2601 Australia; 4 Faculty of Science and Technology, University of Canberra, Kirinari Street, Bruce, ACT 2617 Australia

**Keywords:** *Theba pisana*, *Cernuella virgata*, *Cochlicella acuta*, *Cochlicella barbara*, Estivation, Land snails, Agroecology, Behavioural ecology

## Abstract

Four invasive Mediterranean snails, i.e., *Theba pisana* (Müller, 1774), *Cernuella virgata* (da Costa, 1778), *Cochlicella acuta* (Müller, 1774) and *Cochlicella barbara* (Linnaeus, 1758) cost $170 million yearly to the grain industry in Australia. Their impact is mainly due to their estivation behavior: snails climb on cereal and legume stalks to rest during summer, which coincides with harvest, causing grain contamination issues in crops such as wheat, barley and canola. Diverse management methods have been developed to regulate snail populations, with limited success. Our study investigates the potential for a push–pull strategy to divert invasive snails from cultivated fields. A “push” part (i.e. using a repellent stimuli) was based on the use of a chemical deterrent repelling snails from the cultivated field, and a “pull” part (i.e. using an attractive stimuli) was based on offering attractive estivation supports for snails to aggregate outside the cultivated field. First, artificial estivation supports of different colors were tested under laboratory and field conditions and showed that red supports were the most attractive for these snails. Second, different substances were tested as potential snail deterrents (garlic, coffee, coffee grounds, copper). Garlic extracts were the most powerful snail deterrent and were shown to effectively protect an estivation support and food source from snails under laboratory conditions. These results, which were highly consistent for the four species, illustrate the potential of a push–pull strategy against invasive snails in Australia. It is the first attempt to develop a push–pull strategy relying on both visual and chemical stimuli to achieve results, as well as manipulating the estivation behavior of a pest.

## Introduction

Terrestrial gastropods represent a major threat to agriculture in most temperate and tropical regions^1,2^. The pest status of terrestrial gastropods has increased with crop intensification, the expansion of monocultures, no till and conservation tillage. Numerous introductions of exotic species linked to global trade have also become invasive^3^. Many countries and crop types are affected by these pests. For example, more than 30,000 tons of molluscicides was used to control snail pests on Brassicaceae and ornamental crops in 1994 in France^4^. In the United Kingdom, around £4 million of damage is caused by gastropods yearly on wheat alone^5^. As a result, terrestrial gastropod management costs the agricultural sector millions every year through crop losses and management costs^6,7^.

In Australia, snail and slug management currently costs the grain industry over $170 million each year^8^. A large proportion of these costs is due to the presence of four snail species native to the Mediterranean basin, which have become invasive in Australia and are considered major crop pests: two species with a globular shell, *Theba pisana* (Müller, 1774) (Helicidae) and *Cernuella virgata* (da Costa, 1778) (Hygromiidae), and two species with a conical shell, *Cochlicella acuta* (Müller, 1774) (Hygromiidae) and *Cochlicella barbara* (Linnaeus, 1758) (Hygromiidae)^3^. These snails feed mainly on dead organic matter but can also consume green plant tissue and, in the case of *T. pisana*, can cause damage to emerging crops and pastures^9,10^. The main negative impact of these snails is their estivation behavior. In early summer, snails climb vertical supports, usually plants, to avoid hot soil temperatures. They enter diapause and remain there during the warmest months of the year^11^. The presence of snails on cereal and legume stems (e.g. wheat, barley, canola) causes heavy contamination of grain as their estivation coincides with crop harvest. The presence of snail shells obstructs harvesting machinery and reduces grain quality. In addition, snail mucus can contaminate plants needed for animal grazing^8,12^. Furthermore, these four snail species are of quarantine concerns for importing countries like China. Contamination with these snails can prevent exportation of the crop^13^. Snail control in Australia has been the subject of an integrated pest management program for several decades. Current methods of snail control include the use of molluscicides, such as baits containing carbamate or metaldehyde, as well as physical and mechanical methods: burning vegetation at snail estivation sites and crushing shells with chains or steel rollers^8,9^. However, these methods remain unsatisfactory due to their high cost, limited effectiveness, and, in some cases, their significant impact on non-target species^8,9,14,15^. Biological control has been investigated as well. Snail nematodes have been explored by Charwat and Davies^16^, then later by Wang et al.^17^, but their findings did not lead to the development of new management tools. A dipteran parasitoid, *Sarcophaga villeneuveana* (formally *Sarcophaga penicillata*), was introduced as a classical biological control agent against *C. acuta* in the early 2000s^18,19^. Unfortunately, despite successful establishment of *S. villeneuveana* in Australia, the impact of the parasitoid has not regulated snail populations in the field^20,21^.

To improve the current chemical, biological, and mechanical control methods used against these species, alternative management strategies need to be explored. One such control strategy is the development of a push–pull method. Push–pull strategies use repellent and attractive stimuli, deployed in tandem, to manipulate the distribution of a pest and prevent it from damaging valuable crops. Pests are repelled or pushed away from the resource (push) using stimuli that are deterrent or mask the resource, and are simultaneously attracted (pull), using attractive stimuli, to other areas such as traps or trap crops where the pests concentrate, facilitating their elimination^22^. To reduce the impact of the four invasive snail species on the grain industry, the main challenge is to prevent snails from climbing on cereal stalks at the beginning of the estivation period and prior to the harvest. Understanding how snails choose their estivation supports is therefore critical to use this behavior for management. A push–pull strategy against these snails could combine chemical (push) and visual (pull) methods. The use of visual stimuli in push–pull systems has already been explored in several cases, for instance with the use of UV traps against xylophagous insects^23^ or the use of visually attractive sticky traps to control the cherry fruit fly *Rhagoletis cerasi*^24,25^. A recent study by Hanache et al.^26^ explored the notion that visual stimuli could be exploited to construct artificial estivation supports to facilitate the mass capture of invasive snails. Their laboratory and field experiments showed that snails preferred the tallest and widest supports for estivation. These results suggest that snails have preferences for vertical supports based on visual cues, although vision in terrestrial gastropods is thought to be too poorly developed to see objects clearly^27^. As for the “push” part of the push–pull system, repellent or arrestant substances have clear potential to protect valuable crops. Schüder et al.^28^ demonstrated the repellent properties of garlic against the slug *Deroceras panormitanum*, with a barrier efficacy of 65% and Hollingsworth et al.^29^ highlighted the efficacy of caffeine in killing and repelling *Zonitoides arboreus*, suggesting that these two substances could be used to develop gastropod deterrents.

The aim of this study was to further develop an innovative control method against invasive snails in Australia based on a push–pull method. The first objective of the study was to improve the attractiveness of the artificial estivation supports developed by Hanache et al.^26^, by testing snail preferences for supports of different colors in choice tests under laboratory and field conditions. The second objective was to test different barriers based on substances with repellent properties against snails documented in the literature (coffee, coffee ground, garlic), Snails are known to be more active when humidity is high^30^. It was therefore important to identify a barrier that would keep its efficacy as a barrier once wetted. The most effective treatments under dry conditions were therefore tested again under wet conditions. Finally, the ability of these substances to prevent snails from reaching a food source was evaluated.

## Methods

### Snails

Four species were used in this study: two species with a globular shell, *T. pisana* and *C. virgata*, and two species with a conical shell, *C. acuta* and *C. barbara*. Shell diameter can reach up to 30 mm for *T. pisana*, and 20 mm for *C. virgata*. For the two conical shell species, shell height is < 18 mm for *C. acuta*, and < 10 mm for *C. barbara*. The ecology and behavior of these four species are relatively similar. Snails lay eggs in clusters in the upper soil layer from late autumn to the end of winter, and newly hatched snails feed and grow during winter and spring^3^. As temperatures rise and humidity decreases in early summer, they begin to climb vertical supports, such as plant stems or fence posts, where they remain inactive over summer. Rain during estivation can trigger activity, and snails may move down from their supports for short periods before climbing back and resuming estivation^9,31^.

### Sampling, maintenance, and preparation of snails

Snails were collected from the field every two months between February and August 2023 for experiments. Individuals were collected randomly to form a representative sample of the population. Only individuals larger than 3 mm were collected to facilitate observation during experiments. The four snail species were collected in France, in the Hérault department: *T. pisana* (mean shell diameter 11.6 ± 1.9 mm) was collected near Lunel on a weedy roadside reserve (43.590943, 4.104513), *C. virgata* (mean shell diameter 7.0 ± 1.9 mm) in Mauguio in a grassy verge in a parking lot (43.615150, 4.006284), *C. acuta* (mean shell height 5. 2 ± 1.9 mm) in Saint-Clément-de-Rivière in a weedy road reserve (43.677718, 3.856258) and *C. barbara* (mean shell height 5.2 ± 1.7 mm) in Montferrier-sur-Lez in a weedy grassland (43.684283, 3.875300).

All individuals of the same species were stored in large plastic boxes (39 × 33 × 15 cm) with wet absorbent paper at the bottom. They were sprayed with water, fed regularly with *Sonchus oleraceus* leaves and kept at room temperature (temperature 20 ± 5 °C) under natural photoperiod. The day before each experiment, individuals were selected randomly from these boxes, cleaned, and placed in a separate plastic box containing only *S. oleraceus* leaves as food. A few minutes before the experiment, the snails were sprayed with water to activate them. Snails were discarded after the experiment.

### Test under laboratory conditions

For color strip choice, pole choice and barrier experiments, snails were placed individually in a circular arena with a black self-adhesive film on the floor (“Uni schwarz lack”, d-c-fix®, Weißbach, Germany)^26^ and a white matte self-adhesive film venilia wall (“Uni weiss lack”, d-c-fix®, Weißbach, Germany). This arena had a height of 15 cm for all species and a diameter of 20 cm for conical shell species or 25 cm for globular shell species. Each test was carried out in a controlled environment at 24 ± 1 °C ambient temperature and 53 ± 10% humidity and illuminated with LEDs (flux at 1 m: 100 µmol/m^2^/s).

#### Color strip choice test

For each species, snails were placed individually in the center of the arena so that the anterior end of the snail was facing two 10.5 × 6.4 cm Maya paper strips (270 g/m^2^, Clairefontaine®) of different colors stuck to the arena wall with clear adhesive tape (scotch) and spaced 6.4 cm apart (Supplementary Material, Fig. S1). The test consisted of offering a choice between two colors: a black paper strip and a red, green, purple, orange, blue or yellow paper strip (primary and secondary colors). Black is already known to be attractive to these snails^26,32^ and was used here as a standard to compare with other colors. To detect possible positional biases in this experiment, the position of the color strip (left or right to the black strip) was regularly switched, and a control test with two black strips was carried out. A total of 30 individuals were tested per color pair (i.e., 210 snails per species). It was considered that a snail had made a choice when it touched and climbed onto a strip or remained at the base of a strip. If a snail touched the wall of the arena or did not touch anything at the end of the test, it was considered as a “no choice”. Globular shell species had 10 min to make a choice and conical shell species had 15 min because of their smaller size and slower speed.

#### Color post choice test

For each species, snails were placed individually in the center of the arena and faced 2 colored posts corresponding to black and the color preferred by each species in the color strip choice experiments. These plastic posts were made of high-density polyethylene irrigation pipe, they measured 10.5 × 2.5 cm and were spaced 6.4 cm apart (Supplementary Material, Fig. S2). To detect possible positional biases, the position of the posts was switched after each snail and a control test with two black posts was carried out. A total of 50 snails were tested for the choice of colored posts and the control test (i.e., 100 individuals per species). As for the color test, a snail was considered to have made a choice if it touched and climbed one of the posts within 10 min or 15 min, and a no choice if it didn’t. The time taken to make a choice was recorded.

### Field post choice test

To assess if the laboratory results would be consistent under field conditions, a field experiment was conducted with *T. pisana* and *C. virgata*. There were not enough *C. acuta* and *C. barbara* collected to conduct this experiment. Ten quadrats 50 cm × 50 cm containing four PVC posts 80 cm high and 10.5 cm in diameter were placed in a field site. The experiments were carried out in August during sunny days in a mowed grassland area located near Montpellier (43.684366, 3.874910). Each quadrat was spaced 2 m apart and contained 2 posts covered with black venilia and 2 posts covered with red venilia (“Uni weiss lack”, d-c-fix®, Weißbach, Germany). The posts were placed at each corner of the quadrat and the arrangement of colors was alternated between quadrats (Supplementary Material, Fig. S5). Grass within the quadrats was cut before the experiments to remove other potential estivation support. Each test was carried out as follows: in the late afternoon, 100 snails were placed in the center of each quadrat and were sprayed with water to activate them. Approximately 22 h after the beginning of the test, the number of snails that had climbed onto each post was counted. The number of single snails and aggregated snails (i.e., snails in groups of two or more with their shells touching) were recorded as well as the number of snails in each aggregate. The position of each post within the quadrat was marked to identify potential positional biases. At the end of each test, snails on and off posts were removed, and each post was washed with water to remove traces of mucus. Three replicates of 10 quadrats were done for each species (30 quadrats in total). One replicate was made per day. New snails were used for each test.

#### Dry and wet barrier test

Based on a literature search, the following substances were selected as potential repellents against the four snail species: sand, garlic powder, coffee powder, coffee grounds (35 g of coffee powder to 600 mL water in a coffee maker), and copper strips^28,29^. For each species, snails were placed alone in the center of the arena, facing a red post (10.5 × 2.5 cm). This post was surrounded by a thin layer of the dry substance tested to form a barrier on the floor of the arena. Copper strips were used as a positive control as it has been reported as an effective barrier for snails and is sold to that effect^28,33,34^. Sand was used to determine the repellent effect of the physical aspect of the barrier alone (i.e., dry and grainy aspect of the barrier). Finally, barrier-free tests served as a negative control. Each treatment was applied over a width of 3 cm for globular shell species and 1.5 cm for conical shell species (Supplementary Material, Fig. S3), the width of the barrier being proportional to the size of the shell of the different species^28^. To determine if coffee and garlic treatments retained their efficiency under humid conditions, a second set of tests were carried out, this time moistening the barrier using a water spray bottle. The following treatments were tested: wet garlic, wet coffee, wet coffee grounds, negative control, and dry garlic as positive control. Table 1 summarizes the treatments used as barriers and the quantities applied in each experiment. Treatments were tested immediately after they were wetted.

**Table 1:** All treatments used for dry and wet barrier tests.

Treatment	Barrier test conditions	Quantity of dry matter used for an arena (g) (small arena|large arena)	Quantity of water used to humidify an arena treatment (mL) (small arena|large arena)	Product reference
Purified sand	Dry	0.5|1.5	–	Very fine sea sand, Merck®, No CAS: 14808-60-7
Copper (positive control)	Dry	–	–	Copper Barrier Tape, SureFire products ®
Coffee	Dry and wet	0.5|1	0.1|0.7	Ground coffee Ethiopia Pure Arabica mocha bio, Destination
Garlic	Dry and wet	0.1|0.15	0.1|0.7	Garlic powder bio, Cook, Référence: 3417960022459
Coffee grounds	Wet	3|12	–	Coffee grounds from Ground coffee Ethiopia Pure Arabica mocha bio, Destination
Nothing (Negative control)	Dry and wet	–	–	–

“Small arena” means arena for conical shell snails and “large arena” means arena for globular shell snails.

Thirty individuals were tested for each treatment, i.e., 300 snails per species. Snails were placed in the arena. Whether they crossed the barrier and climbed onto the pole, or remained outside the barrier (either by remaining within the arena or exiting the arena within the test time) was noted. Based on observations from previous experiments, the duration of a test was set at 10 min for *T. pisana* and 15 min for *C. acuta*, *C. barbara* and *C. virgata*. Despite their similar size, *C. virgata* took significantly longer time to make a choice than *T. pisana* in the color post choice experiment (Wilcoxon test: W = 1772, P < 0.001).

#### 24 h barrier test

To determine whether the barriers tested were effective in the longer term and could be used to protect a source of food, another experiment was designed with the barriers copper, coffee, garlic, and the negative control treatment over 24 h with *T. pisana*. Five snails starved for a week were placed in a closed plastic box (24 × 18x10 cm) with four *S. oleraceus* leaves in a floral tube in the center. Substances to be tested were applied over a 3 cm width circle around the floral tube in the same way as the dry and wet barrier experiments (Supplementary Material, Fig. S4). Snails were then left for 24 h in the box under controlled conditions at a temperature of 21 ± 0.2 °C, a humidity of 90 ± 5% and a photoperiod of 15:9 L:D. For each treatment, 24 boxes were tested and each *S. oleraceus* leaf was photographed before and after the experiment to estimate damage due to herbivory. Leaf damage was measured using ImageJ software (version 1.54d)^35^.

### Statistical analysis

To assess the effect of color on snail choice in the color strip and color post experiments, Pearson’s Chi-square tests were used for count data, with the null hypothesis that the distribution between the two choices offered to the snails is equal (α = 0.05). Snails that did not make a choice were excluded from the analyses. For each test, the percentage of participation was calculated as the number of snails that made a choice divided by the total number of snails tested, multiplied by 100. To test if choice time is influenced by the presence of a colored post, a Gamma GLM (Generalized Linear Model) was performed, followed by a type II ANOVA post-hoc test. For the field post choice test, the percentage of snails that climbed on posts in each quadrat was calculated as the number of snails that climbed on posts in a quadrat divided by the total number of snails released in a quadrat, multiplied by 100, and the percentage of snails found aggregated on each quadrat was calculated as the number of snails that were found aggregated on posts in a quadrat divided by the total number of snails that climbed on posts in a quadrat, multiplied by 100. To calculate the choice preference between red and black posts, we used a Pearson’s Chi-square tests for count data, with the null hypothesis that the total number of snails that climbed on black posts and the total number of snails that climbed on red posts is equal (α = 0.05). To compare the efficacy of the different barriers tested, Pearson’s Chi-square tests were used with count data, with the null hypothesis that the distribution of the number of individuals who crossed the barrier was equal between each treatment (α = 0.05). For each barrier test, the percentage barrier effectiveness was calculated as the number of snails that failed to reach the post divided by the total number of snails tested, multiplied by 100. For the 24 h barrier tests, the percentage of leaf damage was calculated by dividing the leaf area after 24 h by the leaf area before 24 h, multiplied by 100. The difference of leaf damage between each treatment was analyzed first with a Kruskal–Wallis Rank Sum test (leaf damage did not follow a normal distribution, Shapiro–Wilk normality test: W = 0.910; P < 0.001) and with a Pairwise Wilcox post-hoc test (α = 0.05). All statistical analyses were carried out using RStudio software (version 4.2.0). Excel (version 2312) and the package ggplot2 on RStudio^36^ were used for plots.

## Results

### Color strip choice test

**Figure 1:**
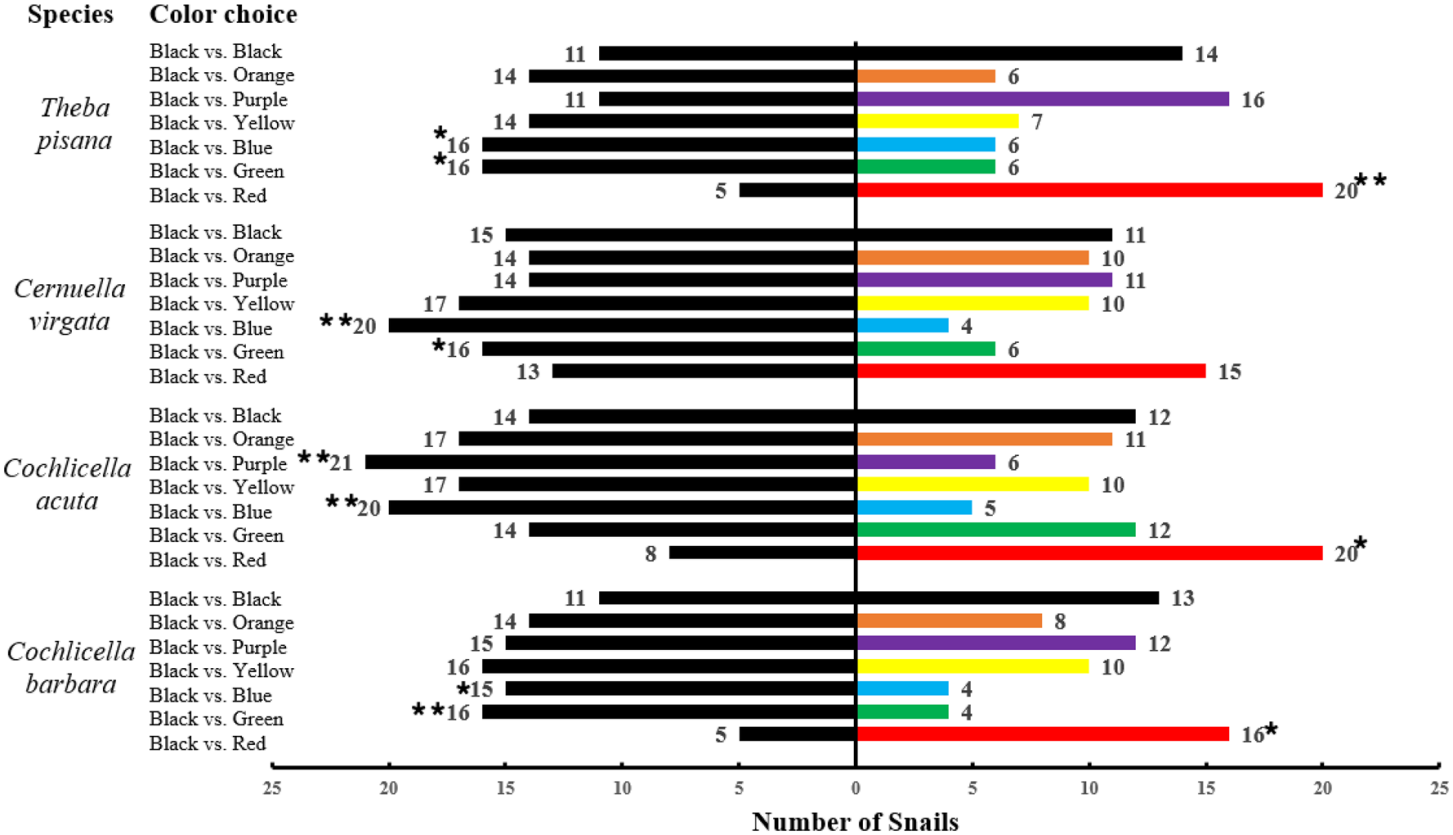
Preference of four snail species for different colors in dual choice-tests under laboratory conditions. Numbers indicate the total number of individuals that made a choice for each color (n = 30 for each color duo). Snails that did not make a choice are not represented. Asterisks indicate significant preference (Chi-square test, α = 0.05; *P < 0.05; **P < 0.01).

*Theba pisana*, *C. acuta* and *C. barbara* showed a significant preference for the red strip compared to the black strip. For *C. virgata*, there was no significant preference for red over black, but this color nevertheless attracted more individuals than the other colors tested (Table 2, Fig. 1). Overall, 70.3 ± 5.8% (Mean ± SE) of individuals chose red over black (total for all four species). Black was significantly more attractive than green and blue in all four species, except for green with *C. acuta*. In total, only 19 snails chose the blue strip out of 120 tested in this color-choice experiment, and 26 chose the green strip. The other colors tested (orange, purple and yellow) showed variable responses across species, but were never preferred over black. The control test showed no significant difference for any of the species. Snail participation was high, with an average of 81.4 ± 1.7% of snails making a choice for all species (Table 2).

**Table 2:** Summary of color strip and color post choice tests under laboratory conditions for (a) *T. pisana* and *C. virgata* and for (b) *C. acuta* and *C. barbara*.

(a) Test category	Color combination	Species									
		*Theba pisana*					*Cernuella virgata*				
		% of choice (n)	χ^2^	P	Significance	% of participation	% of Choice (n)	χ^2^	P	Significance	% of participation
Color choice	Red vs. Black	80.0 (20) vs. 20.0 (5)	**9.000**	**0.003**	**	83.3	53.6 (15) vs. 46.4 (13)	0.143	0.706	NS	93.3
	Green vs. Black	27.3 (6) vs. 72.7 (16)	**4.546**	**0.033**	*	73.3	27.3 (6) vs. 72.7 (16)	**4.546**	**0.033**	*	73.3
	Blue vs. Black	27.3 (6) vs. 72.7 (16)	**4.546**	**0.033**	*	73.3	16.7 (4) vs. 83.3 (20)	**10.667**	**0.001**	**	80.0
	Yellow vs. Black	33.3 (7) vs. 66.7 (14)	2.333	0.127	NS	70.0	37.0 (10) vs. 63.0 (17)	1.815	0.178	NS	90.0
	Purple vs. Black	59.3 (16) vs. 40.7 (11)	0.926	0.336	NS	90.0	44.0 (11) vs. 56.0 (14)	0.360	0.549	NS	83.3
	Orange vs. Black	30.0 (6) vs. 70.0 (14)	3.200	0.074	NS	66.7	41.7 (10) vs. 58.3 (14)	0.667	0.414	NS	80.0
	Black vs. Black	56.0 (14) vs. 44.0 (11)	0.360	0.549	NS	83.3	42.3 (11) vs. 57.7 (15)	0.615	0.433	NS	86.7
Color post choice	Red vs. Black	67.4 (29) vs. 32.6 (14)	**5.233**	**0.022**	*	86.0	62.5 (25) vs. 37.5 (15)	2.500	0.114	NS	80.0
	Black vs. Black	56.4 (22) vs. 43.6 (17)	0.641	0.423	NS	78.0	42.5 (17) vs. 57.5 (23)	0.900	0.343	NS	80.0

(b) Test category	Color combination	Species									
		*Cochlicella acuta*					*Cochlicella barbara*				
		% of choice (n)	χ^2^	P	Significance	% of Participation	% of choice (n)	χ^2^	P	Significance	% of participation
Color choice	Red vs. Black	71.4 (20) vs. 28.6 (8)	**5.143**	**0.023**	*	93.3	76.2 (16) vs. 23.8 (5)	**5.762**	**0.016**	*	70.0
	Green vs. Black	46.2 (12) vs. 53.8 (14)	0.154	0.695	NS	86.7	20.0 (4) vs. 80.0 (16)	**7.200**	**0.007**	**	66.7
	Blue vs. Black	20.0 (5) vs. 80.0 (20)	**9.000**	**0.003**	**	83.3	21.1 (4) vs. 78.9 (15)	**6.368**	**0.012**	*	63.3
	Yellow vs. Black	37.0 (10) vs. 63.0 (17)	1.815	0.178	NS	90.0	38.5 (10) vs. 61.5 (16)	1.385	0.239	NS	86.7
	Purple vs. Black	22.2 (6) vs. 77.8 (21)	**7.000**	**0.008**	**	90.0	44.4 (12) vs. 55.6 (15)	0.333	0.564	NS	90.0
	Orange vs. Black	39.3 (11) vs. 60.7 (17)	1.286	0.257	NS	93.3	36.4 (8) vs. 63.6 (14)	1.636	0.201	NS	73.3
	Black vs. Black	46.1 (12) vs. 53.9 (14)	0.154	0.695	NS	86.7	54.2 (13) vs. 45.8 (11)	0.167	0.683	NS	80.0
Color post choice	Red vs. Black	61.2 (30) vs. 38.8 (19)	2.469	0.116	NS	98.0	59.1 (26) vs. 40.9 (18)	1.455	0.228	NS	88.0
	Black vs. Black	47.7 (21) vs. 52.3 (23)	0.091	0.763	NS	88.0	50.0 (21) vs. 50.0 (21)	0.000	> 0.999	NS	84.0

*N.S* not significant.

For the color strip choice tests, 30 snails were tested per color combination. For the color post choice tests, 50 snails were tested per color combination. Chi-square test, α = 0.05; *P < 0.05; **P < 0.01.

Significant values are in bold.

### Color post choice test

**Figure 2:**
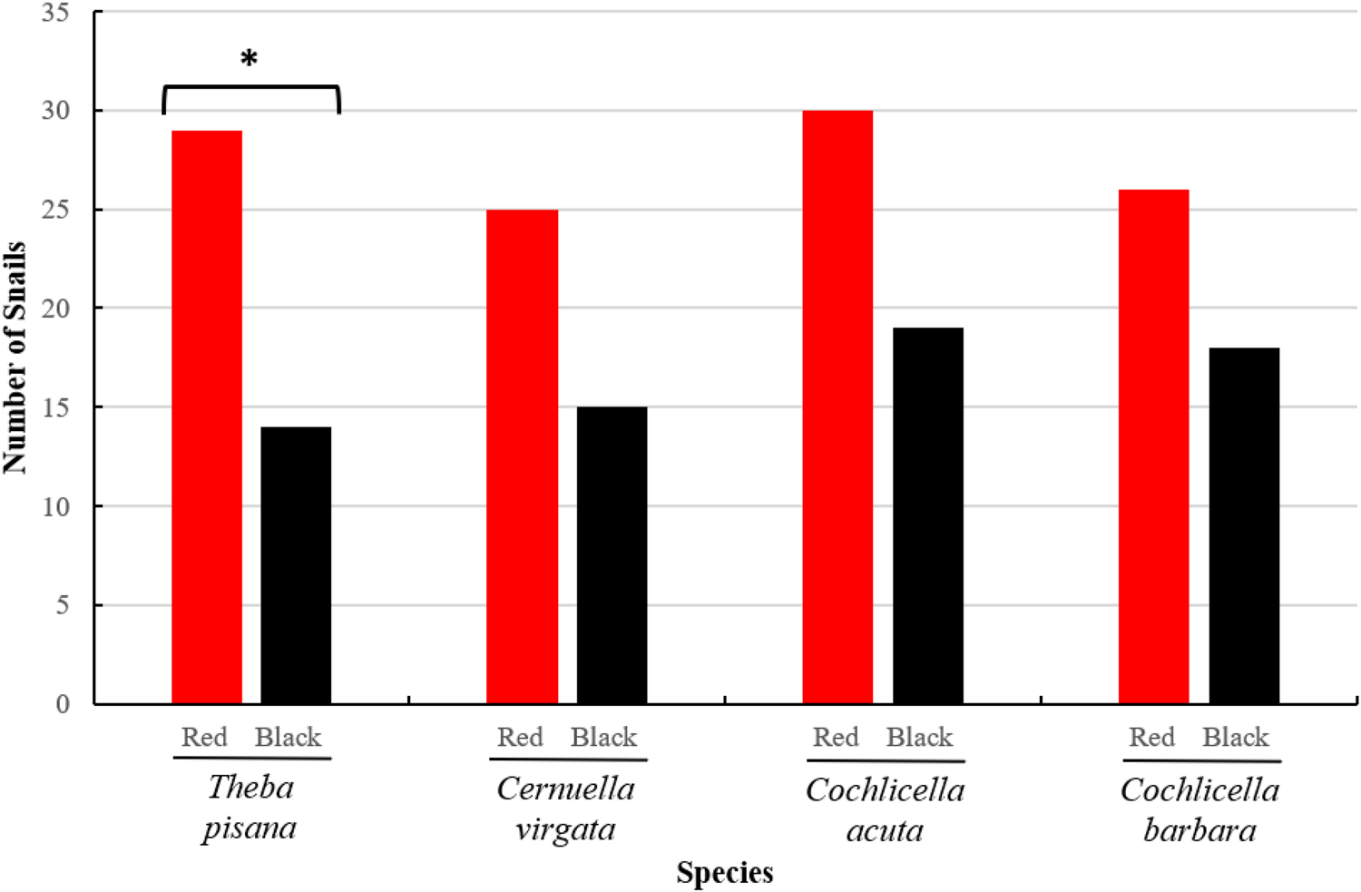
Preference of four snail species for colored post choice tests under laboratory conditions (n = 50 for each species). Snails that did not make a choice are not represented on this graph. Asterisks indicate a significant color preference. Chi-square test, α = 0.05; *P < 0.05.

In total, 110 individuals chose the red post compared to 66 individuals choosing the black post out of 200 snails tested (Chi-square test: χ^2^ = 11, P ≤ 0.001). *Theba pisana* showed a significant preference for the red post over the black post (χ^2^ = 5.233, P = 0.023). The other three species do not show a significant preference for red, but they exhibited the same trend, with an average of 10 more choices for red than black for these three species (Fig. 2, Table 2). Snail choice times between the control and red post tests showed no significant difference (GLM Gamma. *T. pisana*: χ^2^ = 3.731, P = 0.053; *C. virgata*: χ^2^ = 1.041, P = 0.308; *C. acuta*: χ^2^ = 0.691, P = 0.406; *C. barbara*: χ^2^ = 2.273, P = 0.132). The control test showed no significant difference for the four species. All the color post choice tests showed good participation, with an average of 85.3 ± 2.3% (Table 2).

### Field post choice test

**Figure 3:**
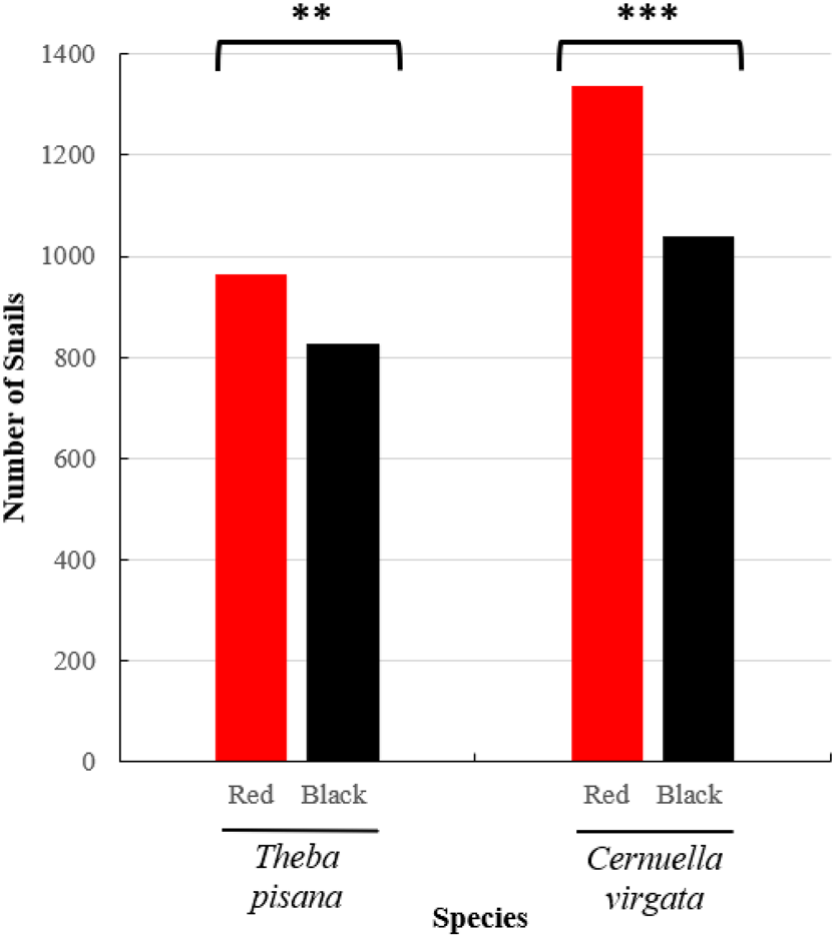
Preference of *T. pisana* and *C. virgata* for colored post choice tests under field conditions (n = 3000 for each species). Snails that did not make a choice are not represented on this graph. Asterisks indicate a significant color preference. Paired T-test, α = 0.05; **P < 0.01; ***P < 0.001.

For the two species tested, the majority of snails placed in the quadrats were found on the posts after 22 h. For *T. pisana*, 59.7 ± 3.0% snails were found on the posts in each quadrat (1791 out of 3000 snails released) and the percentage of snails aggregated was 57.0 ± 2.5% (1055 out of 1791 snails that climbed). For *C. virgata*, 79.3 ± 1.6% (2380 out of 3000 snails released) snails were found on the posts in each quadrat (2380 out of 3000) and the percentage of snails aggregated was 71.4 ± 1.9% (1711 out of 2380 snails that climbed). For the post choice test, contrary to the laboratory experiments, *T. pisana* showed significant preference for red posts (Chi-square test: χ^2^ = 10.176; df = 1; P = 0.001): 963 snails were found on red posts against 828 on black posts. However, *C. virgata* showed a significant preference for red posts (Chi-square test: χ^2^ = 37.313; df = 1; P < 0.001) with 1339 snails found on red posts against 1041 on black posts (Fig. 3).

### Dry and wet barrier tests

**Figure 4:**
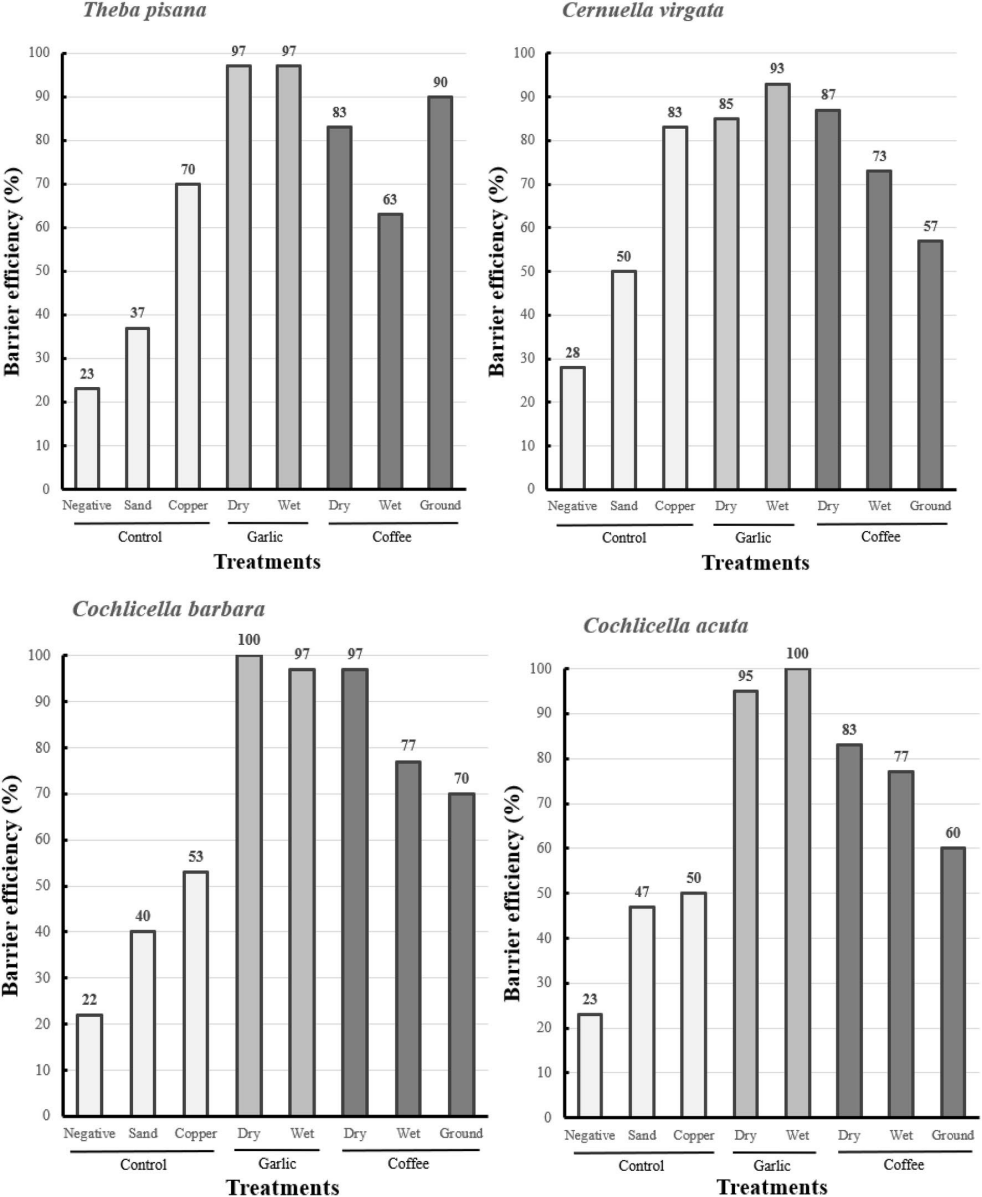
Percentage of barrier efficiency (number of individuals failing to reach the red post divided by the total number of individuals, multiplied by 100) of each treatment for the four snail species. The percentage of negative control and dry garlic treatments have been tested twice. The percentage displayed on the graph for these treatments corresponds to the average efficiency of the dry and wet barrier tests.

In dry barrier experiments, garlic was the most effective barrier stopping 94.2 ± 3.2 of snails overall and 100% of *C. barbara* (Fig. 4). It was also significantly more effective than copper for *T. pisana*, *C. acuta* and *C. barbara* (Table 3). Coffee was also an effective barrier stopping 87.5% ± 3.2% of snails. Coffee was significantly more effective than copper for both conical shell species (Table 3). Copper (positive control) was not as effective on conical shell snails; it had an average efficacity of 76.7 ± 6.7% against the two globular shell species and only 51.7 ± 1.7% for the two conical shell species. There was no significant difference between the negative control and sand for all four species. Sand deterred fewer snails than garlic and coffee for all four species and deterred fewer snails than copper for the two globular shell species (Table 3). The difference in efficacy between copper and sand was 8.3 ± 5.0% for conical shell species, compared with 31.7 ± 1.7% for globular shell species.

**Table 3:**
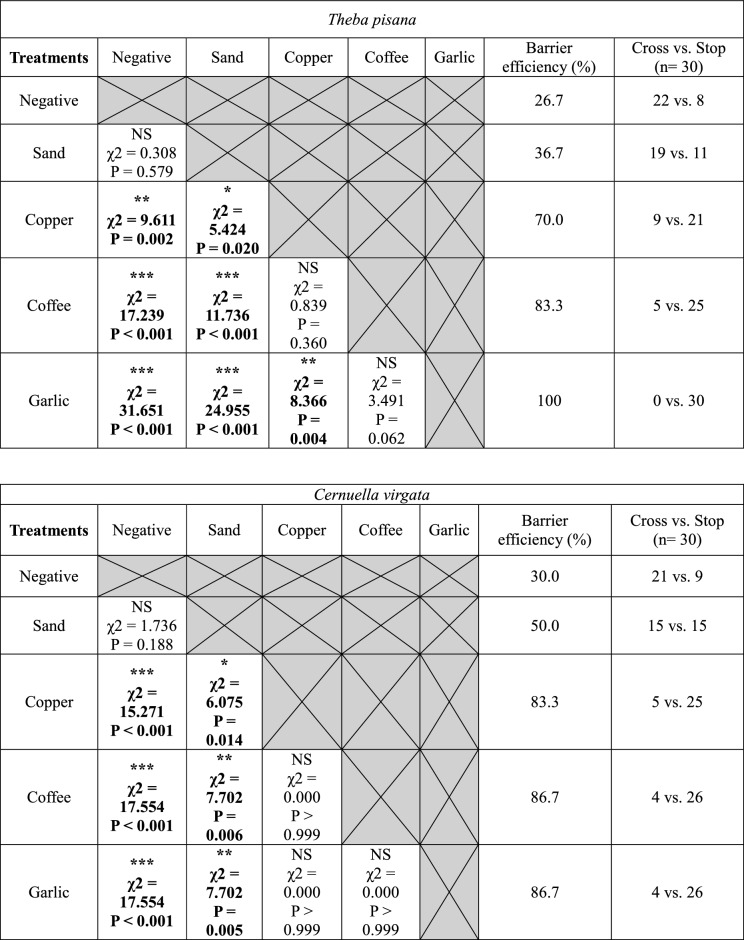

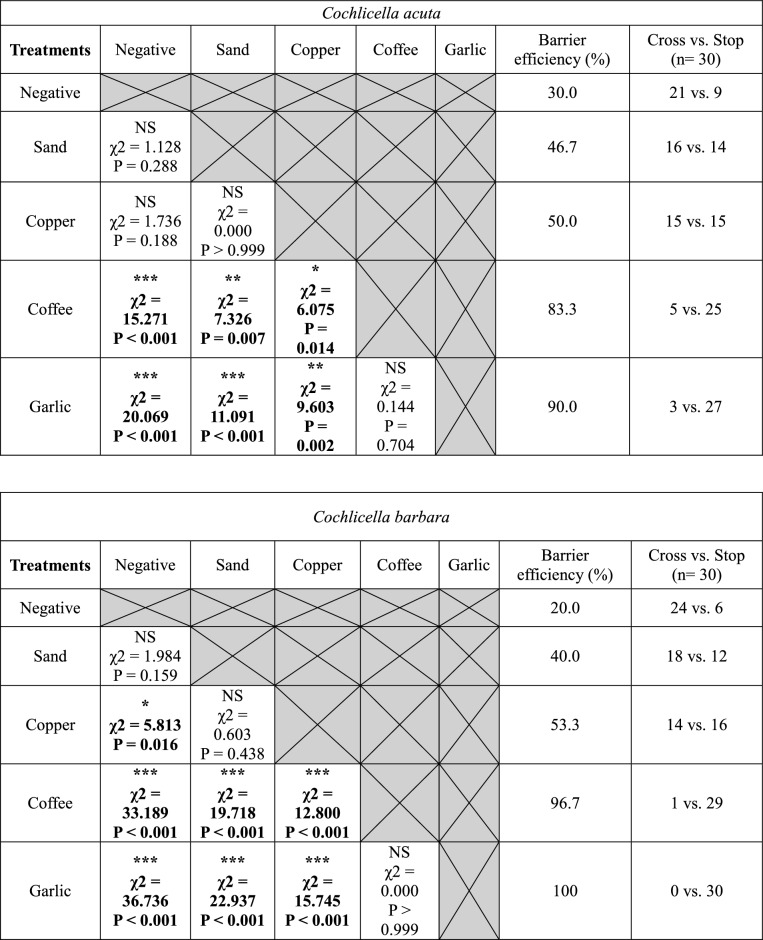
Comparison of the efficacy of the barriers tested presented as Chi-square test matrices between the different treatments used in the dry barrier experiments for the four snail species.

Negative = negative control, Sand = physical control; Copper = positive control.

“Cross” corresponds to the number of snails that reached the post and “Stop” corresponds to the number of snails that did not reach the post. Asterisks indicate a significant difference between two types of treatment (α = 0.05; *P < 0.05; **P < 0.01; ***P < 0.001. *N.S* not significant).

In the wet barrier experiments, wet garlic was as effective as dry garlic and stopped most snails from reaching the posts. Moistened coffee was less effective than dry coffee. Coffee lost an average of 15.0 ± 3.2% efficacy when moistened, but this difference was not significant for any of the four species (Fig. 4, Table 4). On average, coffee grounds were 69.2 ± 7.5% effective. For conical shell species, coffee grounds were as effective as wet coffee, but significantly less effective than wet garlic (Table 5). Coffee grounds were more effective than wet coffee for *T. pisana*, but not for *C. virgata*. Dry and wet garlic were significantly more effective than coffee grounds for *C. virgata* (Table 5). All treatments used in the wet barrier test were significantly more effective than the negative control for all four species, with increased efficacy of 70.0 ± 2.7% for wet garlic, 45.8 ± 4.2% for wet coffee and 42.5 ± 8.6% for coffee grounds compared with the negative control.

**Table 4:** Comparison of dry and wet barrier tests for garlic, coffee, and coffee grounds under laboratory conditions for (a) *T. pisana* and *C. virgata* and for (b) *C. acuta* and *C. barbara*.

(a)	*Theba pisana*				*Cernuella virgata*			
Treatments (dry vs. wet)	Cross/stop (dry treatment) vs. cross/stop (wet treatment)	χ^2^	P	Significance	Cross/stop (dry treatment) vs. cross/stop (wet treatment)	χ^2^	P	Significance
Dry garlic vs. wet garlic	0/30 vs. 3/27	1.404	0.236	NS	4/26 vs. 2/28	0.185	0.667	NS
Dry coffee vs. wet coffee	5/25 vs. 11/19	2.131	0.144	NS	4/26 vs. 8/22	0.938	0.333	NS
Dry coffee vs. ground coffee	5/25 vs. 3/27	0.144	0.704	NS	4/26 vs. 13/17	**5.253**	**0.022**	*

(b)	*Cochlicella acuta*				*Cochlicella barbara*			
Treatments (dry vs. wet)	Cross/stop (dry treatment) vs. cross/stop (wet treatment)	χ^2^	P	Significance	Cross/stop (dry treatment) vs. cross/stop (wet treatment)	χ^2^	P	Significance
Dry garlic vs. wet garlic	3/27 vs. 0/30	1.404	0.236	NS	0/30 vs. 1/29	0.000	> 0.999	NS
Dry coffee vs. wet coffee	5/25 vs. 7/23	0.104	0.747	NS	1/29 vs. 7/23	3.606	0.058	NS
Dry coffee vs. ground coffee	5/25 vs. 12/18	2.955	0.086	NS	1/29 vs. 9/21	**5.88**	**0.015**	*

*N.S* not significant.

“Cross” corresponds to the number of snails that reached the post and “Stop” corresponds to the number of snails that did not reach the post. Dry garlic treatments have been tested twice, in this table “Dry garlic” is the result from the dry barrier experiments. 30 snails were tested for each type of treatment. Chi-square test, α = 0.05; *P < 0.05.

Significant values are in bold.

**Table 5:**
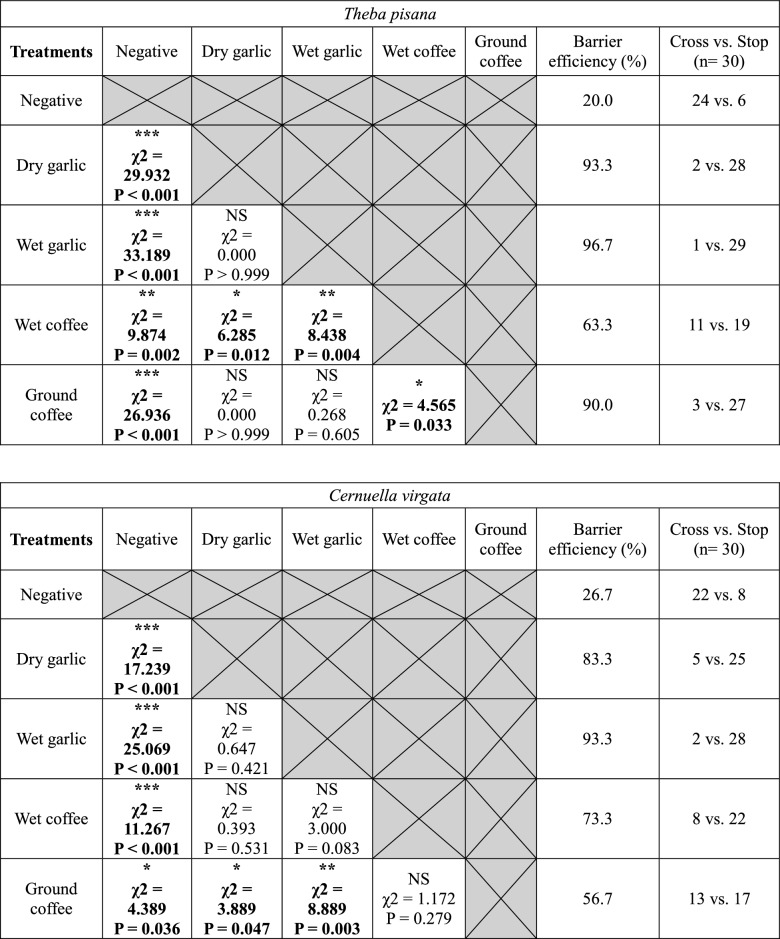

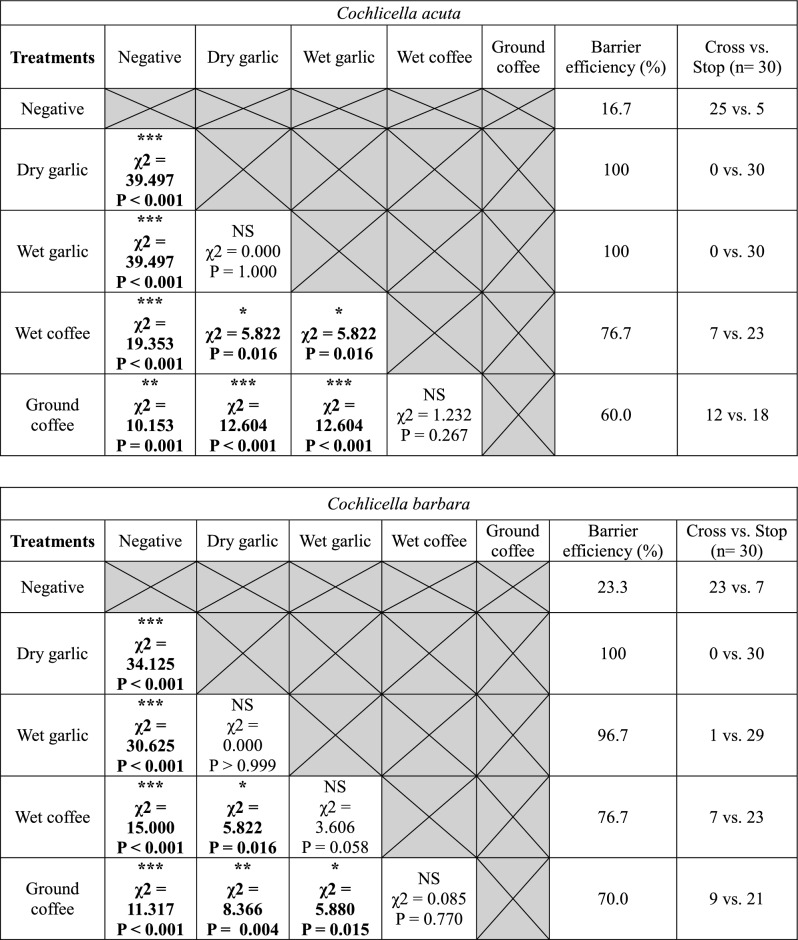
Chi-square test matrices between the different treatments used in the wet barrier experiments for the four snail species.

Negative corresponds to the negative control and dry garlic corresponds to the positive control.

“Cross” corresponds to snails that reached the post and “Stop” corresponds to the number of snails that did not reach the post. Asterisks indicate a significant difference between two types of treatment (α = 0.05; *P < 0.05; **P < 0.01; ***P < 0.001. *N.S* not significant).

### 24 h barrier test

**Figure 5:**
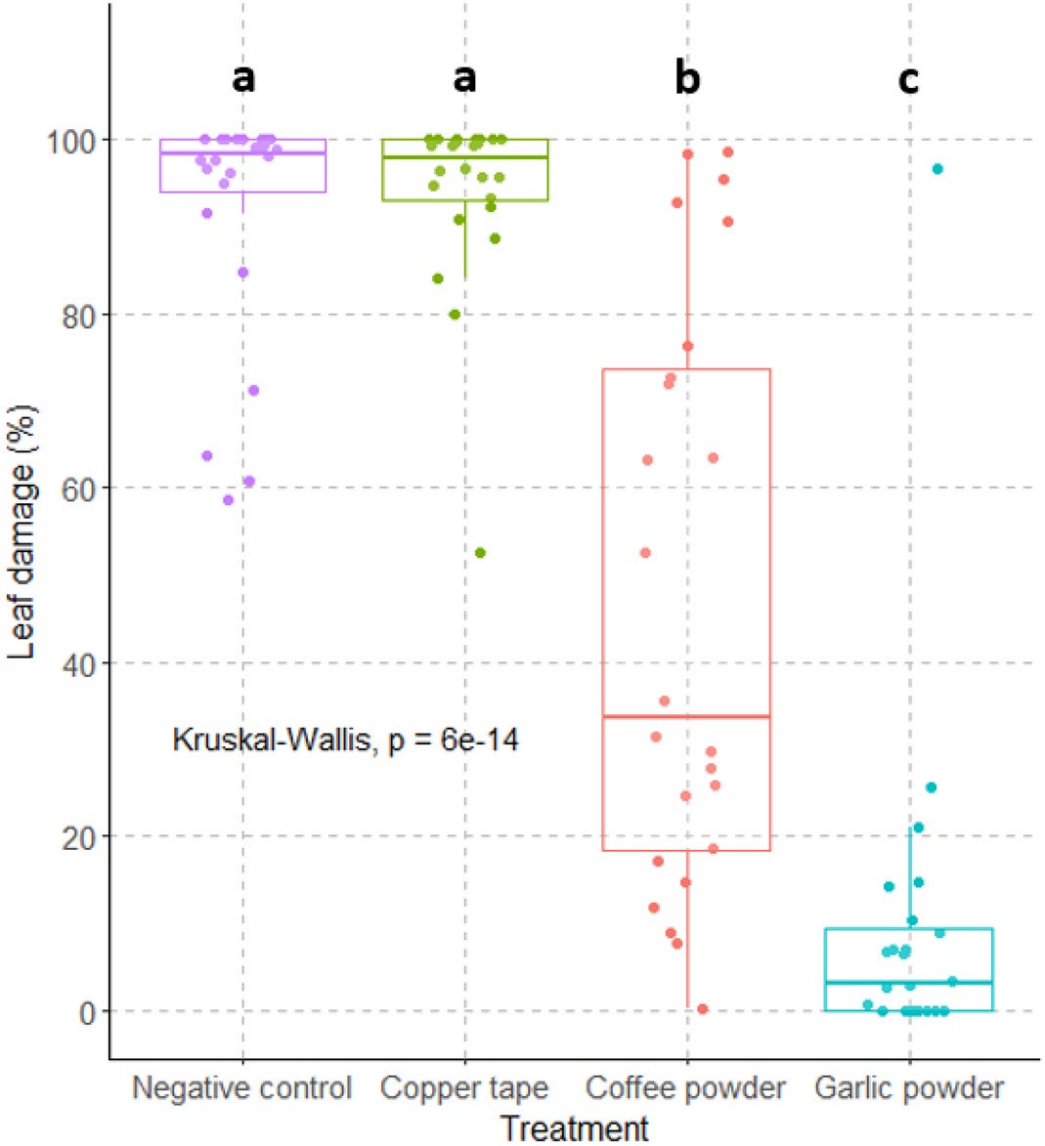
Leaf damage of *S. oleraceus* leaves for each treatment in 24 h barrier test for *T. pisana*. Means with a different letter are statistically different (Kruskal–Wallis test and Pairwise Wilcox post-hoc test: α = 0.05).

After the 24 h barrier tests for *T. pisana*, the percentage of leaf damage was significantly different among treatments (Kruskal–Wallis test: χ^2^ = 64.629, df = 3, P ≤ 0.001). The average leaf damage of *S. oleraceus* for the garlic treatment was 9.5 ± 4.1%. Leaf damage was only present in 6 out of 24 replications. With the coffee treatment, the average leaf damage was 47.1 ± 6.8% and damage to *S. oleraceus* leaves was present in 14 replications out of 24. The negative treatment had significantly more leaf damage than the garlic and coffee treatments (P < 0.001), but no significant difference was observed with the copper treatment (P > 0.99) (Fig. 5). Both the negative control and the copper treatment had visible leaf damage in all 24 replications (with 92.0 ± 2.8% of leaf damage for the negative treatment and 94.1 ± 2.1% of leaf damage for the copper treatment).

## Discussion

The development of new tools to reduce the impact of terrestrial gastropods in agriculture is a worldwide challenge, particularly in Australia where invasive snails cost the grain industry over $170 million each year^8^. Our study describes the foundations of an innovative push–pull strategy based on attraction to visual stimuli and repellence by chemical barriers, which could be applied to the periphery of fields to protect crops from snails invading fields. Artificial supports of red color were the most attractive to snails under laboratory and field conditions. Garlic was the most efficient barrier tested and was shown to protect a support or a food source from snails under laboratory conditions. Promisingly, these results were consistent for the four species of invasive snails.

The use of attractive stimuli in integrated pest management for terrestrial gastropods has centered mainly on food baits with palatable plants^37,38^, but our study proposes a new approach based on visual stimuli. Deploying colored vertical supports around fields could facilitate mass capture of snails during the estivation period and prevent snails from reaching crops. Our results show that snails can differentiate between different colored strips under controlled conditions, and that their preferences for colored strips are consistent with their choices of colored estivation supports. *Theba pisana*, *C. acuta* and *C. barbara* showed a marked preference for the red color during color strip and post choice tests. Moreover, field test with *T. pisana* and *C. virgata* also showed an overall preference for red, confirming laboratory results.

These results raise questions about the visual capabilities of these snails. The general consensus on snail vision is that the eye of terrestrial gastropods cannot produce sharp images of objects but can still distinguish between the overall distribution of light and darkness. It allows snails to orient towards dark areas^39,40^. Our choice-test results suggest that snails do not simply orient toward the darkest visible point in their environment, otherwise black would have been consistently preferred to all the other colors tested. Black is already known to be attractive to snails^26,32^. However, the preference for the red color in terrestrial gastropods had not yet been documented, and red does not play an obvious role in the ecology of the four species studied (i.e. food source coloration, secondary sexual characteristics, etc.). The preferences observed in our color tests may be explained by contrast differences with the background. Indeed, several studies have shown that red and black offer the best contrast with natural backgrounds. For example, Lee et al.^41^ and Schmidts et al.^42^ showed that red and black fruits were significantly more contrasted with foliage and bark than other fruit color groups, making them more conspicuous to frugivorous bird species. Color choices based on contrasts also emerged in investigations of visual stimuli in the fly *Rhagoletis pomonella*, where several colors, including red and black were more attractive for this species^43^. The author hypothesized that this fly preferred these colors because they contrasted most with the foliage background and other reflected or transmitted light rather than showing true color discrimination. Gastropods do not have the ability to see colors, but their ability to distinguish the distribution of light and darkness could allow them to distinguish colors based on achromatic contrast, i.e. the difference in the intensity between the light reflected from the whitest part of an image and from the blackest part^44^. Contrast-based choices would also explain why blue and green were chosen less often by snails in our experiments, since these two colors were relatively bright and offered little contrast with the white background of the experimental arena. Furthermore, changes in background and brightness between laboratory and field conditions may explain why support choices for *T. pisana* and *C. virgata* were not fully consistent between laboratory and field tests. It is also important to note that red and black do not always differ from each other in terms of contrast with natural backgrounds^41,42^, which could explain why there were sometimes no significant differences during the choice tests for these two colors.

Barrier tests highlighted potential new repellents against invasive snails in Australia. Garlic, and to a lesser degree, coffee were effective at preventing snails from reaching vertical supports and food sources under laboratory conditions. Garlic powder was highly effective against the four species studied even after moistening. Coffee also stopped many snails, but lost effectiveness once moistened. This may explain why results of the 24 h barriers tests with coffee were very heterogeneous, as humidity was very high within experimental boxes (84.5 ± 0.4%). Moreover, results showed that coffee grounds prevented the majority of *T. pisana* and *C. barbara* from reaching vertical supports (90% and 70% barrier efficacy, respectively). However, this result is unlikely to be due to a repellent effect. Indeed, the four species were seen to move freely on coffee grounds and *T. pisana* was observed consuming coffee grounds rather than reaching vertical supports. Therefore, coffee grounds do not seem to be an effective repellent for these snails and may even represent a food source.

Copper strips were shown to prevent the two globular shell species from reaching vertical supports, although they were less effective than garlic or dry coffee. However, copper appeared to be much less effective against the two conical shell species tested. Copper is commonly used against terrestrial gastropods in home gardens and agriculture, but studies on its efficacy have only focused on slug species or large snails^28,45,46^. It is therefore possible that static electricity, which is thought to be the basis of the repulsive effects generated by copper^47^, affects small snail species to a lesser extent. In addition, during the 24 h barriers tests, the high humidity level within experimental boxes may have changed the static electricity potential of copper strips. Tests with sands showed that a dry, granular substance is not in itself a barrier to snails. The repellent properties of garlic and coffee demonstrated in our experiments are thus likely to be based on their chemical composition rather than their physical texture.

One candidate molecule that could explain the repellent effect of garlic is allicin. Allicin is an organosulfur compound found in certain species of the Liliaceae and Alliaceae families. This molecule is a secondary compound with anti-bacterial and antifungal properties, and it also plays a role in defense against phytophagous insects^48^. Allicin is already known in pest control for its repellent properties on crop pests. Debra and Misheck^49^ achieved significantly higher survival of their cabbage from insect pests by mixing their crop with garlic plants. Toxic effects of allicin were observed on the beetles *Tribolium castaneum*, *Oryzaephilus surinamensis* and *Cryptolestes ferrugineus*, with an 85% to 100% reduction in pupal emergence depending on the species^50^. Our results, along with those of Schüder et al.^28^ on the slug *Deroceras panormitanum* and the snail *Oxyloma pfeifferi,* suggest that garlic has real potential for management of terrestrial gastropods.

The use of coffee in pest management is poorly documented in the literature^51^. Only Hollingsworth et al.^29^ proposed a caffeine-based solution to control terrestrial gastropods. In our experiments, the repellent properties of coffee may be due to the presence of caffeine, but also to the presence of a high concentration of tannins, which can act as antifeedants for herbivores^52,53^. However, results with coffee barriers were highly variable and provided limited protection of a food source in the 24 h barrier tests, suggesting that this treatment would not be efficient in the field. Coffee grounds appeared to have lost much of their deterrent effect compared to coffee powder, probably due to a lower caffeine content.

Although the results of our study are promising, many questions have yet to be investigated to push further the development of push–pull technology as an applicable management tool against invasive snails in Australia. It is important to remember that the experiments took place in France, so the attractiveness of artificial supports needs to be investigated directly under Australian environmental conditions with local snail populations. While it is possible that Australian snails are different to their native counterparts in France, considering the relative consistency of our results for the four species, it is unlikely that there would be large intraspecific variability when so little interspecific differences were observed in France.

Field experiments in Australia will need to include tests under different environmental conditions and crop types, and include density of artificial support needed to be effective. In our field tests, artificial supports attracted 69.5% of the snails in 24 h in 50 × 50 cm quadrats. More tests would be needed to determine optimal densities of artificial supports depending on the level of snail attractiveness desired. This would also need to be tested within the different crops to ensure snails prefer the artificial support to plant stalks. One key issue regarding the implementation of artificial supports is their cost effectiveness. Indeed, deploying and monitoring artificial supports takes time and money, particularly if snails need to be harvested from posts to be destroyed. The costs associated with this method may quickly surpass its benefits if high densities of posts are needed to protect a field or reduce snail populations meaningfully. Practicality and costs will need to be thoroughly investigated during the development of any field-ready product. In parallel, it remains to be investigated whether garlic, the most deterrent substance identified in our tests, could be effective in the field with weather conditions and if an application at the edge of the crop is sufficient to prevent field contamination from the periphery. Garlic also needs to be developed into a product applicable at a large scale and effective under different environmental conditions.

In a global context where the impact of invasive species worldwide keeps rapidly increasing and where reliance on chemical control draws major environmental concerns, it is urgent and crucial to develop novel pest management strategies that are both effective and safe for the environment. Our study provides a proof of concept for an innovative push–pull strategy against invasive snails in Australia. Attractants (colored vertical supports) and repellents (chemical barriers) could be used in combination to manipulate snail orientation in cultivated fields and reduce their impact on grains without detrimental impact on non-target organisms. Despite their importance as agricultural pests, the ecology of terrestrial snails has been vastly underexplored compared to other groups. Our results that focused on the visual preferences of snails add valuable knowledge to the sensory ecology of terrestrial gastropods and demonstrate that a better fundamental understanding of the physiology, ecology, and behavior of pests can open novel perspectives for their management.

### Supplementary Information

Supplementary Figures.

## Data Availability

The data that support this study is available on DRYAD (10.5061/dryad.c2fqz61hf).
