# Supplementary material for: Toward a push–pull strategy against invasive snails using chemical and visual stimuli
Source: Sci Rep. 2024 May 20;14:11511. doi: 10.1038/s41598-024-62225-6 (PMC11106284; doi:10.1038/s41598-024-62225-6)
Supplement: Supplementary file 1 — Supplementary Figures. [file 41598_2024_62225_MOESM1_ESM.docx]

**Supplementary material**

**Toward a push-pull strategy against invasive snails using chemical and visual stimuli.**

Scientific Reports

Cédric Kosciolek^1^, Gaylord A. Desurmont^2^, Thierry Thomann^1^, Alberto Zamprogna^1^, Valérie Caron^3^*

^1^CSIRO European Laboratory, 34980 Montferrier-sur-Lez, France

^2^European Biological Control Laboratory, USDA-ARS, 810 Avenue de Baillarguet, 34980 Montferrier sur Lez, France

^3^CSIRO Health and Biosecurity, Clunies Ross Street, Acton Australian Capital Territory 2601 Australia

^4^University of Canberra, Faculty of Science and Technology, Kirinari Street, Bruce Australian Capital Territory 2617, Australia

*Corresponding author: [valerie.caron@csiro.au](mailto:valerie.caron@csiro.au)


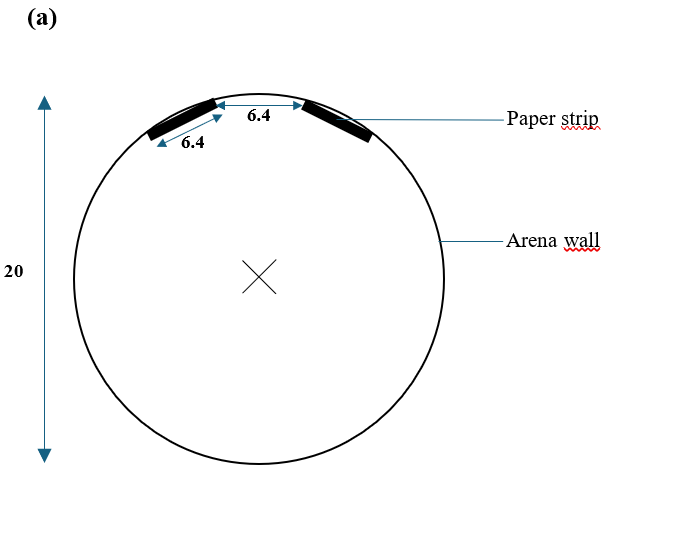

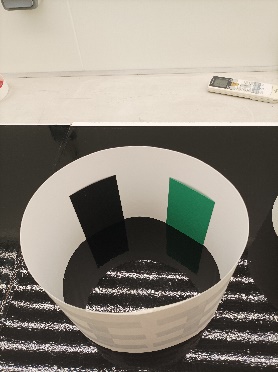


**Fig. S1** Experimental scheme and picture of laboratory tests for color strip choice test for globular shell species. The crosse represents a snail. All numbers are in centimeters. For conical shell species, the arenas were 20 cm wide.


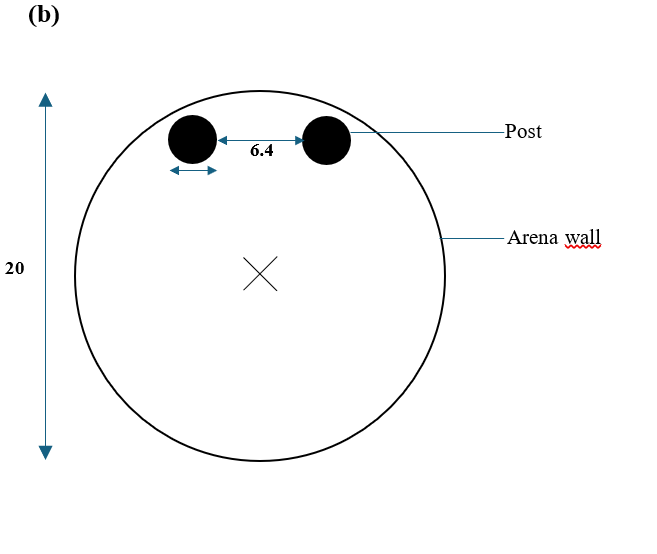


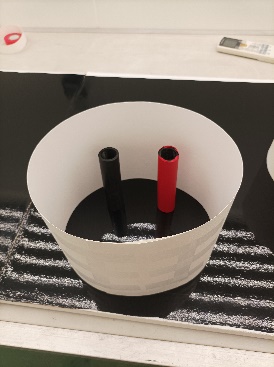


**Fig. S2** Experimental scheme and picture of laboratory tests for Color post choice test for globular shell species. The crosse represents a snail. All numbers are in centimeters. For conical shell species, the arenas were 20 cm wide.


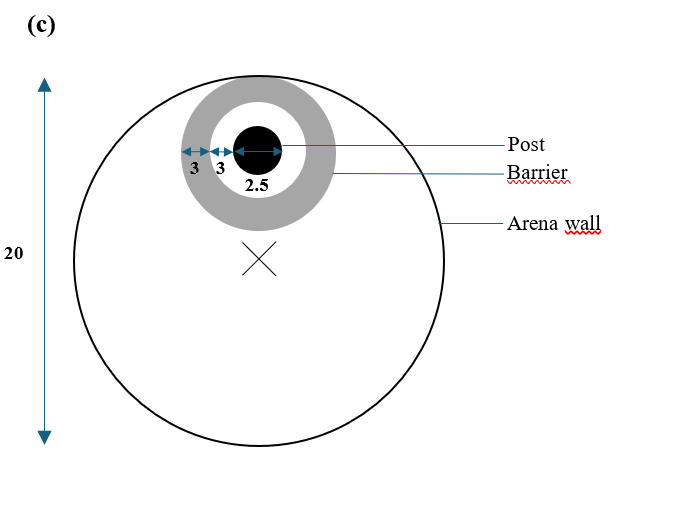


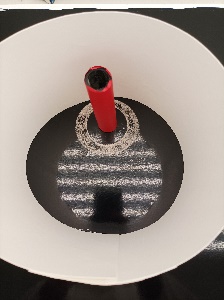


**Fig. S3** Experimental scheme and picture of laboratory tests for dry and wet barrier test for globular shell species. The crosse represents a snail. All numbers are in centimeters. For conical shell species, the arenas were 20 cm wide and the width of the barrier was 1.5 cm.


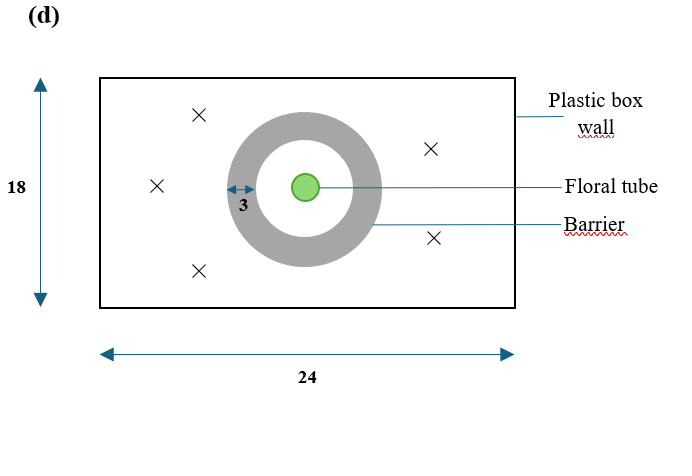


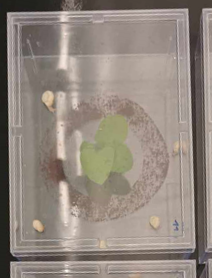


**Fig. S4** Experimental scheme and picture of laboratory tests for 24h barrier test for globular shell species. The crosse represents a snail. All numbers are in centimeters.


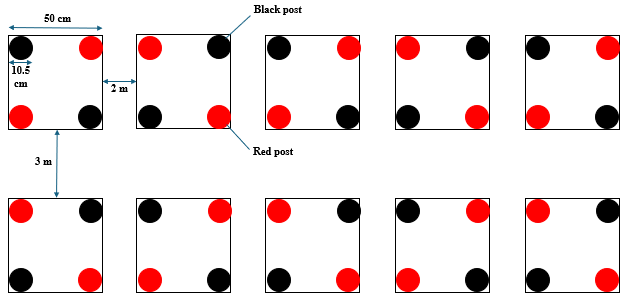


**Fig. S5** Experimental scheme of Field post choice test.
